# Antibiotic Resistance and Virulence Profiles of *Klebsiella pneumoniae* Strains Isolated From Different Clinical Sources

**DOI:** 10.3389/fcimb.2021.738223

**Published:** 2021-09-01

**Authors:** Victoria Ballén, Yaiza Gabasa, Carlos Ratia, Raquel Ortega, Marc Tejero, Sara Soto

**Affiliations:** ISGlobal, Hospital Clínic—Universitat de Barcelona, Barcelona, Spain

**Keywords:** *Klebsiella pneumoniae*, antimicrobial resistance, virulence, biofilm, urinary tract infections

## Abstract

*Klebsiella pneumoniae* is a Gram-negative bacterium capable of colonizing, invading, and causing infections in different anatomical sites of the human body. Its ability to evade the immune system, its increasing antimicrobial resistance and the emergence of hypervirulent pathotypes have become a major challenge in the medical field. In this study, 127 strains from different clinical sources (urine, respiratory tract or blood) were characterized for antimicrobial resistance, the presence of virulence factor genes, serum resistance, hypermucoviscosity and the ability to form biofilms. Specific characteristics of the uropathogenic strains were examined and compared with the other clinical groups.

Differences were found between urine and the other groups of strains. Urine strains showed the highest antibiotic resistance (64.91%) compared to blood (63.64%) or respiratory strains (51.35%) as well as the highest extended-spectrum beta-lactamases (ESBL) production. These strains also showed statistically significant high resistance to fosfomycin (24.56%) compared to the other groups (p = 0.008). Regarding virulence, 84.21% of the urine strains presented the *uge* gene, showing a statistically significant difference (p = 0.03) compared to the other clinical sources, indicating a possible role of this gene in the development of urinary tract infection. In addition, 46% of biofilm-forming strains belonged to the urine sample group (p = 0.043). In conclusion, *K. pneumoniae* strains isolated from urine samples showed higher antimicrobial resistance, ESBL production, and biofilm-forming ability compared to those isolated from respiratory or blood samples. The rapid spread of clinical strains with these characteristics is of concern, and new therapeutic alternatives are essential to mitigate their harmful effects.

## Introduction

*Klebsiella pneumoniae* is an opportunistic pathogen associated with a variety of infections, being urinary tract infections (UTIs) among the most common worldwide. *K. pneumoniae* is considered an important uropathogen in ambulatory patients (Foxman, 2010). Although UTIs do not result in a high mortality rate, they represent a significant economic burden to the health care system as they increase treatment costs.

However, *K. pneumoniae* is not only responsible for UTIs but also for respiratory and bloodstream infections (Podschun and Ullmann, 1998). It is one of the species recognized as part of the ESKAPE group, associated by their characteristic potential to escape or evade the action of antimicrobial agents. This acronym comprises six highly virulent and antibiotic-resistant bacterial pathogens including *Enterococcus faecium*, *Staphylococcus aureus*, *K. pneumoniae*, *Acinetobacter baumannii*, *Pseudomonas aeruginosa*, and *Enterobacter* species. Additionally, the World Health Organization (WHO) lists *K. pneumoniae* as one of the species of high priority and promotes the research and development of new antibiotics due to the growing global problem of antimicrobial resistance (World Health Organization, 2017).

Uropathogenic bacteria have some characteristics that favor the colonization of human cells, such as the production of adhesins, siderophores, and toxins (Foxman, 2010). In the same way, these bacteria can adhere to medical devices forming biofilms, avoiding the immune system and favoring the antimicrobial therapy failure (Dybowska-Sarapuk et al., 2017). For this reason, the characterization of *K. pneumoniae* antimicrobial resistance, its ability to form biofilm and its virulence are key to understanding the pathogenicity of this bacteria in the clinical setting and to achieve more efficient antimicrobial therapy.

The aim of this study was to characterize a collection of *K. pneumoniae* strains isolated from different clinical sources (urine, respiratory tract, or blood) in terms of antimicrobial resistance, the presence of virulence factor genes, serum resistance, hypermucoviscosity, and the ability to form biofilms. The specific characteristics of the uropathogenic strains were studied and compared to *K. pneumoniae* strains isolated from respiratory and/or blood samples.

## Materials and Methods

### Bacterial Strains

One hundred twenty-seven *K. pneumoniae* clinical strains from four Catalonian hospitals (Hospital Clinic de Barcelona, Hospital Universitario de Bellvitge, Hospital del Mar, and Hospital Universitario Mutua de Terrassa) were collected over six months from 2016 to 2017. Among these, 57 strains were obtained from urine (1 urinary catheter, 1 suprapubic aspiration, and 55 midstream urine), 37 from respiratory samples (7 sputum, 10 bronchoalveolar aspirates, 4 tracheal samples, 1 bronchoalveolar lavage, 15 non-classified respiratory samples), and 33 strains were obtained from blood. Strains were identified as *K. pneumoniae* using matrix-assisted laser desorption ionization–time-of-flight mass spectrometry (MALDI-TOF/MS) (Bruker Daltonik GmbH, Bremen, Germany). The modified score values suggested by the manufacturer were used: A score ≥2.3 meant species identification; a score between 2.0 and 2.299 meant genus identification and probable species identification; a score between 1.7 and 1.9 meant probable genus identification; and a score <1.69 meant non-reliable identification. Strains with a score ≥2.3 classified as *K. pneumoniae* were included in the study. The strains were stored in skim milk (BD) at -80°C. All data on the strains used in this study can be found in the **Supplementary Material**.

### Determination of Antimicrobial Resistance

Determination of antimicrobial resistance was assessed by disk diffusion or broth microdilution (in the case of colistin) methods following the Clinical and Laboratory Standards Institute guidelines (CLSI, 2020). The antimicrobial agents tested by Kirby-Bauer method were amoxicillin/clavulanate (20/10 µg), aztreonam (30 µg), cefepime (30 µg), ceftazidime (30 µg), chloramphenicol (30 µg), ciprofloxacin (5 µg), fosfomycin (200 µg/50 µg of glucose-6-phosphate), gentamicin (10 µg), imipenem (10 µg), piperacillin/tazobactam (100/10 μg), and trimethoprim-sulfamethoxazole (1.25/23.75 µg). *E. coli* ATCC 25922 was used as quality control.

Strains were classified according to their antimicrobial resistance profile as susceptible, resistant to 1 or 2 antimicrobial categories, multidrug-resistant (MDR), extensively drug-resistant (XDR) or pandrug-resistant (PDR). The European Centre for Disease Prevention and Control (ECDC) proposed the MDR, XDR, and PDR definitions. An isolate is considered MDR if it is non-susceptible to at least 1 agent in ≥3 antimicrobial categories, defined as XDR if the isolate is non-susceptible to at least 1 agent in all but 2 or fewer antimicrobial categories, and PDR if the isolate is non-susceptible to all listed antimicrobial agents (Magiorakos et al., 2012).

Extended-spectrum beta-lactamases (ESBL) production was evaluated by the ESBL test and carbapenemase production was analyzed by modified carbapenem inactivation and EDTA-modified carbapenem inactivation methods following CLSI guidelines (CLSI, 2020).

### String Test

The string test was performed to identify the hypermucoviscous (HMV) phenotype. The strains were grown in BD Columbia agar with 5% sheep blood (Beckton Dickinson) at 37°C overnight. Afterwards, a bacteriological inoculation loop was used to stretch a mucous bacterial colony. The HMV phenotype was positive when a string > 5 mm in length was observed (Fang et al., 2004).

### Determination of Virulence and Antimicrobial Resistance Genes

A single colony from an overnight culture plate was selected and suspended in 100 μL of sterilized Milli-Q water. The suspension was boiled for 10 min and centrifuged for 10 min at 13000 rpm. The supernatant was used as the DNA template.

Polymerase chain reaction (PCR) was performed to detect different virulence genes: fimbriae (*fimD, fimH, mrkC, mrkD*), capsule-associated genes (*ycfM*, *wabG*, *uge* and *rmpA)*, siderophores (*entB, iucA, irp2*, *ybtS*, *fyuA, iroN*), colibactin genes (*clbA, clbQ*), and capsular serotypes (K1, K2).

ESBL-associated genes *bla*_SHV_, *bla*_TEM_, *bla*_CTX-M-1_, *bla*_OXA48_, in addition to genes conferring resistance to quinolones [*aac (6)-Ib-cr*, *qnrB*], aminoglycosides (*aadb*), and sulfonamides (*sul1* and *sul2*), were detected by PCR. The primers used are listed in **Table 1**.

**Table 1:** Primers to detect virulence and antimicrobial resistance genes.

Target gene	Primer sequence	Melting temperature	Size of amplicon (bp)	Reference
	(5′ → 3′)	(Tm °C)		
*fimD* – F	GTTACGCCTATCTGAATCTACAGAG	57	1114	This study
*fimD* – R	GACCAGTTGATATCGTCCACG			
*fimH* – F	GAAAAAAATAATCCCCCTGTTCAC	57	855	This study
*fimH* – R	GTAACCTGGCCTGTGGTC			
*mrkC* – F	GCTCAACTCCATCGTCAGC	57	1056	This study
*mrkC* – R	CGCGAGTTATTGAACGAGGTG			
*mrkD* – F	CGCTTTTTATCGTCTTAATG	55	880	This study
*mrkD* – R	GTGATGTAGCGGGTCTCCTG			
*ycfM –* F	ATCAGCAGTCGGGTCAGC	57	160	(Candan and Aksöz, 2015)
*ycfM –* R	CTTCTCCAGCATTCAGCG			
*entB* – F	ATTTCCTCAACTTCTGGGGC	60	371	(Candan and Aksöz, 2015)
*entB –* R	AGCATCGGTGGCGGTGGTCA			
*iucA* – F	AATCAATGGCTATTCCCGCTG	58	239	(Garza-Ramos et al., 2018)
*iucA* – R	CGCTTCACTTCTTTCACTGACAGG			
*irp2* – F	GCTACAATGGGACAGCAACGAC	58	230	(Garza-Ramos et al., 2018)
*irp2 –* R	GCAGAGCGATACGGAAAATGC			
*ybtS* – F	GACGGAAACAGCACGGTAAA	53	242	(Compain et al., 2014)
*ybtS* – R	GAGCATAATAAGGCGAAAGA			
*fyuA* – F	TTTCCACCAACACCATCCAG	58	817	This study
*fyuA* – R	CAGGTCAGGTCACTGTATGC			
*iroN* – F	AACCGCCAGATCGATATTCG	58	827	This study
*iroN* – R	TTAATCTCACCGCTGGTTCG			
*rmpA* – F	CATAAGAGTATTGGTTGACAG	56	461	(Compain et al., 2014)
*rmpA* – R	CTTGCATGAGCCATCTTTCA			
*wabG* – F	CGGACTGGCAGATCCATATC	58	683	(Jian-li et al., 2017)
*wabG –* R	ACCATCGGCCATTTGATAGA			
*uge –* F	GATCATCCGGTCTCCCTGTA	59	535	This study
*uge –* R	TCTTCACGCCTTCCTTCACT			
*K1 –* F	GGTGCTCTTTACATCATTGC	59	1283	(Lin et al., 2014)
*K1 –* R	GCAATGGCCATTTGCGTTAG			
*K2 –* F	GACCCGATATTCATACTTGACAGAG	59	641	(Lin et al., 2014)
*K2 –* R	CCTGAAGTAAAATCGTAAATAGATGGC			
*clbA –* F	CTAGATTATCCGTGGCGATTC	51	1002	(Morgan et al., 2019)
*clbA –* R	CAGATACACAGATACCATTCA			
*clbQ* – F	CTTGTATAGTTACACAACTATTTC	50	821	(Morgan et al., 2019)
*clbQ –* R	TTATCCTGTTAGCTTTCGTTC			
*bla*SHV-1 – F	ATGCGTTATATTCGCCTGTG	49	747	(Wiegand et al., 2007)
*bla*SHV-1 – R	TGCTTTGTTATTCGGGCCAA			
*bla*TEM-1 - F	TCGCCGCATACACTATTCTCAGAATGA	53	445	(Monstein et al., 2007)
*bla*TEM-1 - R	ACGCTCACCGGCTCCAGATTTAT			
*bla*CTX-M1 - F	AAAAATCACTGCGCCAGTTC	52	415	(Woodford et al., 2006)
*bla*CTX-M1 - R	AGCTTATTCATCGCCACGTT			
*bla*OXA48 - F	TTGGTGGCATCGATTATCGG	57	744	(Queenan and Bush, 2007)
*bla*OXA48 - R	GAGCACTTCTTTTGTGATGGC			
*aac(6’)-Ib-cr* - F	TTGCGATGCTCTATGAGTGGCTA	60	482	(Park et al., 2006)
*aac(6’)-Ib-cr* - R	CTCGAATGCCTGGCGTGTTT			
*qnrB* – F	GATCGTGAAAGCCAGAAAGG	52	469	(Robicsek et al., 2006)
*qnrB* – R	ACGATGCCTGGTAGTTGTCC			
*aadb* – F	ATGGACACAACGCAGGTCGC	55	534	(Guo et al., 2016)
*aadb* – R	TTAGGCCGCATATCGCGACC			
*sul*1 - F	CTTCGATGAGAGCCGGCGGC	63	436	(Guerra et al., 2004)
*sul*1 - R	GCAAGGCGGAAACCCGCGCC			
*sul*2 - F	TCAACATAACCTCGGACAGT	55	707	(Guerra et al., 2004)
*sul*2 - R	GATGAAGTCAGCTCCACCT			

### Biofilm Formation and Quantification

Biofilm formation was performed using the protocol described by O’Toole and Kolter, (1998) with some modifications. Briefly, isolates were cultured in Luria Bertani (LB) agar (Miller’s LB AGAR, Condalab) for 24 h at 37°C. Then, a single colony of each strain was grown in 10 mL of LB broth (LB, Condalab) for 24 h at 37°C with shaking at 180 rpm. Afterwards, overnight cultures were diluted 1:100 in fresh medium supplemented with 0.25% glucose, 200 µL were inoculated in polystyrene microtiter plates (Nunc™ Edge 2.0 96-well plate, non-treated, with lid, VWR International), and incubated for 48 h at 37°C in static conditions. Culture medium without inoculum was used as a sterility control.

After incubation, the liquid of each well was removed, washed once with 1x phosphate-buffered saline (PBS), and dried at 65°C. Plates were then stained with crystal violet (CV) (2% v/v) for 10 min at room temperature. Next, plates were washed once with 1x PBS and fixed at 65°C for 60 min. The CV was detached by the addition of 33% glacial acetic acid and biomass was calculated measuring the optical density (OD) at 580 nm using a microplate reader (EPOCH 2 microplate reader; BioTek, VT). The experiment was carried out in three technical and biological replicates.

The strains were classified according to the criteria of Stepanović et al. (2007) as: non-biofilm formers (OD ≤ 0.150), weak biofilm formers (≥ 0.151 OD ≤ 0.300), moderate biofilm formers (≥ 0.301 OD ≤ 0.60), or strong biofilm formers (OD ≥ 0.601).

### Serum Resistance

Serum resistance was analyzed following the procedure described by (Podschun et al., 2016). Briefly, 25 µL of inoculum of 2.5 x 10^6^ colony forming units (CFU)/mL of a mid-log phase culture were mixed with 75 µL of normal human serum obtained from healthy volunteers in 96-well polystyrene, round-bottomed microtiter plates (Greiner bio-one). The mixture was incubated at 37°C for 1, 2, and 3 h. After incubation, the cell count was determined using 10-fold serial dilutions and conventional plating in LB agar (Miller’s LB AGAR, Condalab). The test was graded as shown in **Table 2**. The assay was performed in triplicate.

**Table 2:** Classification of serum resistance.

	Cell counts/mL (compared to the original inoculum)		
	1 h	2 h	3 h
**Grade 1**	<10%	<10%	<0.1%
**Grade 2**	10% - 100%	10% - 100%	<10%
**Grade 3**	>100%	<100%	<100%
**Grade 4**	>100%	>100%	<100%
**Grade 5**	>100%	>100%	>100% (decreasing)
**Grade 6**	>100%	>100%	>100% (increasing)

### Statistical Analysis

Statistical analysis was performed using the Chi-square test with the IBM SPSS Statistics software for Windows, version 21.0. *P*-values < 0.05 were considered statistically significant.

## Results

### Antimicrobial Resistance

**Figure 1:**
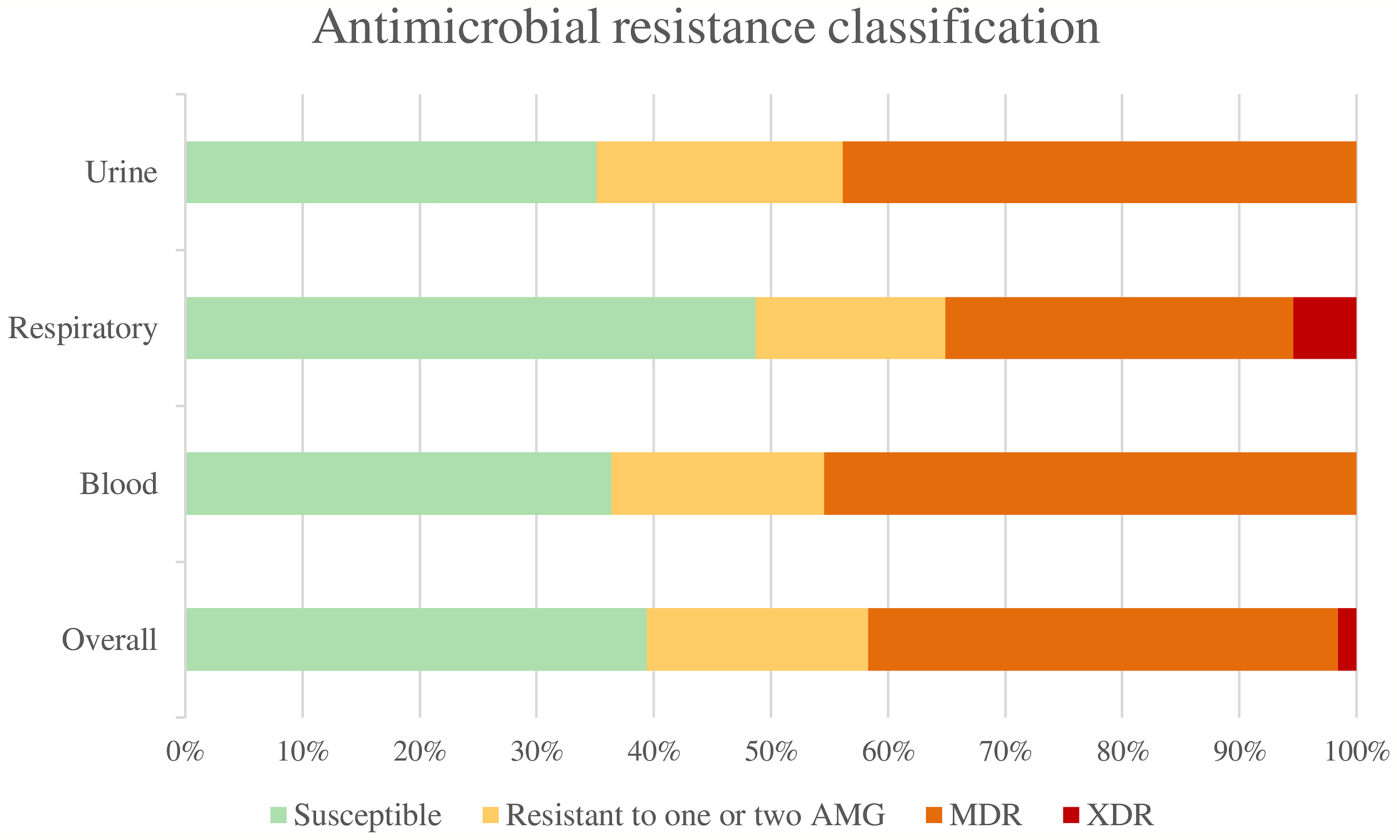
Antimicrobial resistance classification. AMG, Antimicrobial groups; MDR, multidrug-resistant; XDR, extensively drug-resistant.

**Figure 2:**
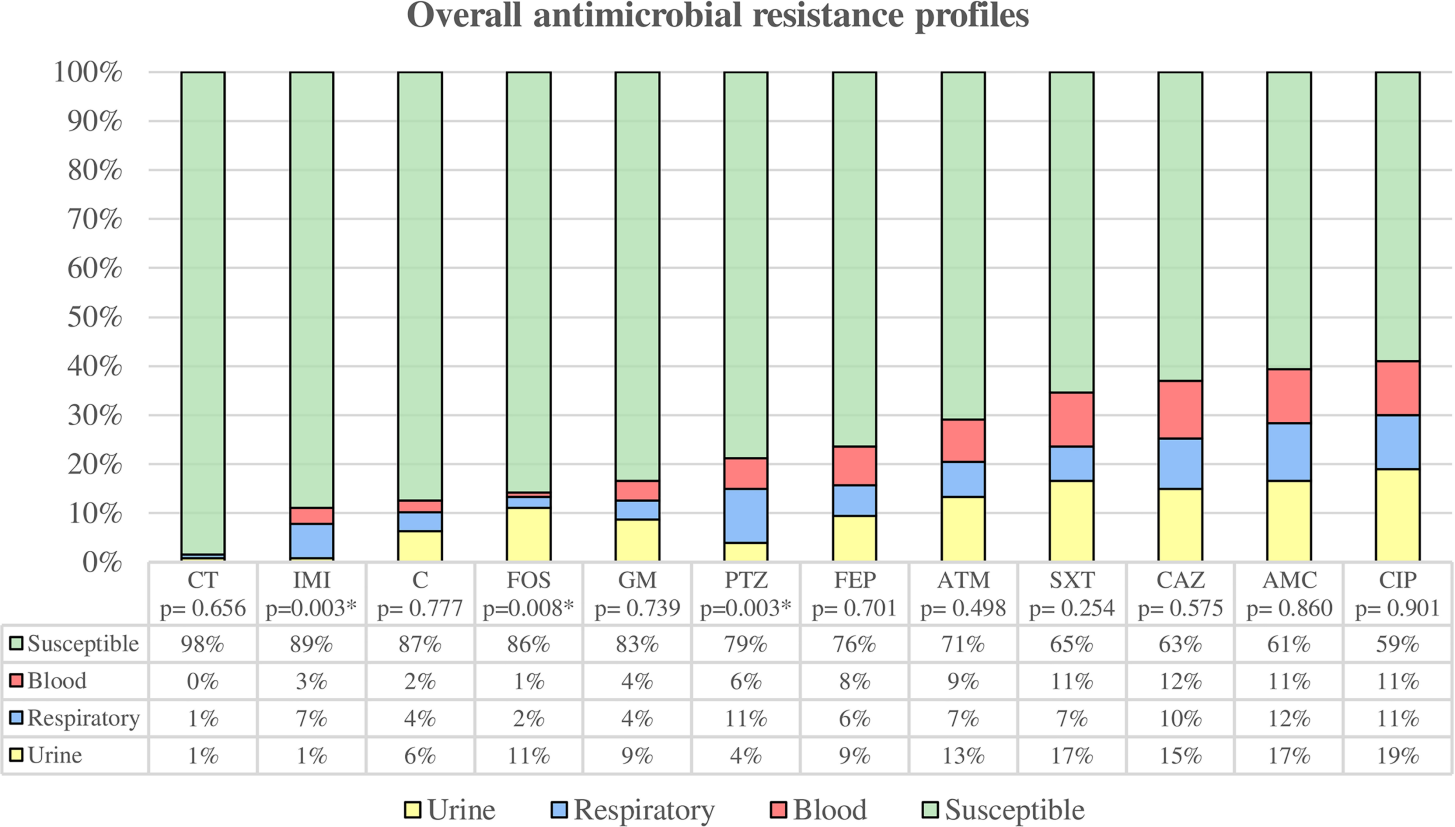
Overall antimicrobial resistance profiles were categorized by clinical source. Susceptibility rate includes all clinical sources, while resistance rates are classified by urine, respiratory tract, and blood. * Statistically significant (p < 0.05). CT, colistin; IMI, imipenem; C, chloramphenicol; FOS, fosfomycin; GM, gentamicin; PTZ, piperacillin/tazobactam; FEP, cefepime; ATM, aztreonam; SXT, trimethoprim-sulfamethoxazole; CAZ, ceftazidime; AMC, amoxicillin/clavulanate; CIP, ciprofloxacin.

Overall, 50 isolates (39.37%) were susceptible to all the antimicrobial agents tested, 24 isolates (18.90%) were non-susceptible to 1 or 2 categories of antibiotics, 51 isolates (40.16%) were considered multidrug-resistant (MDR), and 2 strains (1.57%) were considered extensively drug-resistant (XDR). None was classified as pandrug-resistant (**Figure 1**). The percentages of resistance for each antibiotic tested are shown in **Figure 2**.

Among uropathogenic *K. pneumoniae*, we found 20 susceptible strains (35.09%), 12 strains (21.05%) were non-susceptible to 1 or 2 categories, and 25 were MDR strains (43.86%). The uropathogenic group showed the highest overall antibiotic resistance (64.91%) compared to blood (63.64%) and respiratory tract (51.35%) strains, although these differences were not statistically significant. The highest percentages of antimicrobial resistance were observed for ciprofloxacin (42.11%), amoxicillin/clavulanic acid (36.84%), trimethoprim-sulfamethoxazole (36.84%) and ceftazidime (33.33%). Uropathogenic *K. pneumoniae* also showed the highest resistance to fosfomycin (24.56%) with the difference being statistically significant (p = 0.008) compared to the other groups.

Among the respiratory group, 18 strains (48.65%) were classified as susceptible, 6 strains (16.22%) as non-susceptible to 1 or 2 categories, 11 strains (29.73%) as MDR, and 2 strains (5.40%) as XDR. The highest percentages of antimicrobial resistance were observed with amoxicillin/clavulanic acid (40.54%), ciprofloxacin (37.84%), piperacillin/tazobactam (37.84%) and ceftazidime (35.14%). In comparison to the other groups, respiratory strains showed the highest resistance to imipenem (24.32%) and piperacillin/tazobactam (37.84%), being these differences statistically significant (p = 0.003 both).

Strains isolated from blood samples were classified as follows: 12 strains (36.36%) were susceptible, 6 strains (18.18%) were non-susceptible to 1 or 2 categories, and 15 strains (45.46%) were MDR. The highest percentages of antimicrobial resistance were observed with ceftazidime (45.45%), followed by ciprofloxacin, amoxicillin/clavulanic acid and trimethoprim-sulfamethoxazole with a percentage of resistance of 42.42% each.

### Presence of Antimicrobial Resistance Genes

Fifty-five isolates (43.31%) were ESBL producers. Among these, 24 (43.6%) were isolated from urine, 16 (29.1%) from blood, and 15 (27.3%) from the respiratory tract. The gene most frequently detected was *bla*_SHV-1_ (85.45%), followed by *bla*_CTX-M1_ (78.95%), and *bla*_TEM-1_ (71.93%). Thirteen strains (10.24%) were carbapenemase producers: 9 from the respiratory tract (69.2%), 4 from blood (30.8%) and none from urine. Among these, 10 (76.92%) harbored the *bla*_OXA48_ gene. Concerning the 52 (40.94%) strains resistant to ciprofloxacin, 20 (38.46%) harbored the *aac (6’)-Ib-cr* gene, and 27 (51.92%) harbored the *qnrB* gene. One strain among the group of gentamicin-resistant strains harbored the *aadb* gene. Regarding the 44 strains classified as resistant to trimethoprim-sulfamethoxazole (34.65%), six (13.64%) and 29 (65.91%) carried the *sul1* and the *sul2* genes, respectively.

### Presence of Virulence Genes

**Figure 3:**
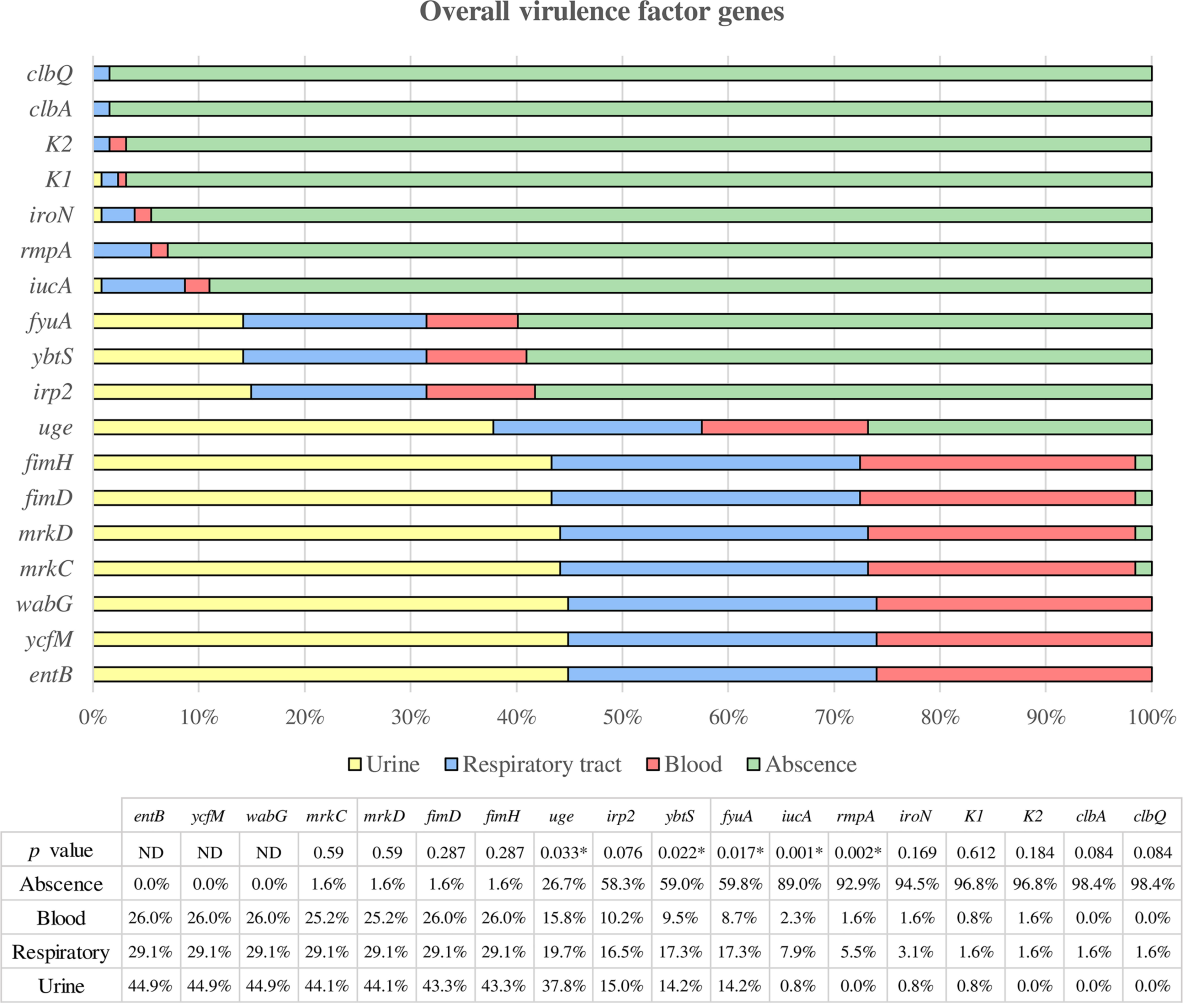
Overall virulence factor genes were categorized according to the clinical source. All strains in which the respective gene is not present are assigned to the “absence” category, while the presence of the gene is classified according to the clinical source (urine, respiratory and blood). * Statistically significant (p < 0.05). ND, Not determined.

The results obtained are shown in **Figure 3**. All the isolates carried the *ycfM*, *entB*, and *wabG* genes. The *fimD*, *fimH*, *mrkC*, and *mrkD* genes were almost ubiquitous among the strains (98.43%). The prevalence of the other virulence genes was as follows: *uge* (73.23%), *irp2* (41.73%), *ybtS* (40.94%), *fyuA* (40.16%), *iucA* (11.02%), *rmpA* (7.09%), *iroN* (5.51%), *clbA* (1.57%), and *clbQ* (1.57%).

Among all these genes, the *uge* gene showed a higher prevalence among urinary strains compared to respiratory and blood samples, being the difference statistically significant (p = 0.033). On the other hand, the *iucA*, *ybtS*, *fyuA* and *rmpA* genes were statistically more prevalent in respiratory isolates than urinary or blood strains (p = 0.001, p = 0.022, p = 0.017, and p = 0.002, respectively).

### String Test and Serum Resistance

In the present study, 17 *K. pneumoniae* strains (13.39%) were identified as hypermucoviscous by the string test. Among these, 7 strains were isolated from urine, 7 from the respiratory tract and 3 from blood.

**Table 3** shows the results of serum resistance. We observed that more than 50% of the strains tested were highly resistant to serum, but there was no correlation between this test and the other characteristics under study (type of isolate, biofilm formation, hypermucoviscosity, antimicrobial resistance classification, or the presence of virulence genes).

**Table 3:** Results of serum resistance test.

	Serum resistance			
	Urine	Respiratory	Blood	Overall
	57 (44.88%)	37 (29.13%)	33 (25.99%)	127 (100%)
**Grade 1**	18 (31.58%)	7 (18.92%)	6 (18.18%)	31 (24.41%)
**Grade 2**	6 (10.53%)	6 (16.22%)	3 (9.09%)	15 (11.81%)
**Grade 3**	3 (5.26%)	1 (2.70%)	0 (0%)	4 (3.15%)
**Grade 4**	1 (1.75%)	0 (0%)	0 (0%)	1 (0.79%)
**Grade 5**	2 (3.51%)	0 (0%)	1 (3.03%)	3 (2.36%)
**Grade 6**	27 (47.37%)	23 (62.16%)	23 (69.70%)	73 (57.48%)

### Biofilm Formation and Quantification

**Figure 4:**
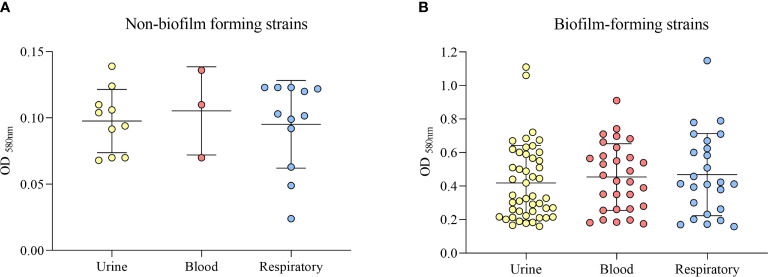
Classification of biofilm formation ability by the clinical source (urine, blood, or respiratory tract). **(A)** Non-biofilm-forming strains. **(B)** Biofilm-forming strains.

Twenty-five strains (19.69%) were classified as non-biofilm formers, 35 (27.55%) as weak biofilm formers, 42 (33.07%) as moderate biofilm formers, and 25 strains (19.69%) as strong biofilm formers. Among the 102 biofilm-forming strains, including weak, moderate and strong, 47 (46.1%) were from urine, 30 (29.4%) were from blood, and 25 strains (24.5%) were from the respiratory tract (**Figure 4**). When grouping the strains into biofilm-forming and non-biofilm-forming, we found that the highest number of biofilm-forming strains were from urine, and compared to the other groups of isolates, the difference was statistically significant (p = 0.043).

### Relationship Between Antimicrobial Resistance and Virulence

There was a statistically significant relationship between the strains carrying the *iucA*, *rmpA* and *uge* genes and the strains classified as susceptible in the antimicrobial resistance classification (p = 0.028; p = 0.006; p < 0.001).

Excluding colistin, we observed a statistically significant relationship between the presence of the *uge* gene and the susceptibility to the other antibiotics tested (p < 0.005). Likewise, strains harboring the *rmpA* gene were more susceptible to amoxicillin/clavulanate, ciprofloxacin, trimethoprim-sulfamethoxazole and cefepime (p = 0.012; p = 0.010; p = 0.023; p = 0.046). Strains harboring *iroN* or *iucA* genes were more susceptible to amoxicillin/clavulanate (p = 0.028; p = 0.042) and ciprofloxacin (p = 0.023; p = 0.032). In addition, strains carrying *iroN* genes were also more susceptible to trimethoprim-sulfamethoxazole (p = 0.048).

A similar relationship was found between the strains carrying the *irp2* gene and susceptibility to ceftazidime and gentamicin (p = 0.020; p = 0.040), and the strains harboring the *ybtS* gene and the susceptibility to gentamicin (p = 0.009).

The *irp2*, *ybtS*, and *fyuA* genes showed a higher prevalence among ESBL-producing strains (p < 0.001, p = 0.02, and p = 0.012, respectively), and the *rmpA* and the *uge* genes were more prevalent among the non-ESBL-producing strains (p = 0.043 and p < 0.001, respectively). Nevertheless, the *irp2, ybtS* and *uge* genes showed a higher prevalence among non-carbapenemase-producer strains (p = 0.034; p = 0.029; p = 0.003).

Concerning the hypermucoviscous phenotype, the lack of the *iucA*, *iroN*, and *rmpA* genes was related to the non-hypermucoviscous phenotype (p < 0.05 in all cases). Hypermucoviscous strains also showed a lower capacity to form biofilm (p = 0.017) and a higher susceptibility to gentamycin and amoxicillin/clavulanate (p = 0.049 both).

There were significant differences between biofilm‐forming and non-biofilm‐forming isolates concerning the *iucA*, *iroN*, and *rmpA* genes, with biofilm-forming strains lacking these genes (p = 0.02, p = 0.01, and p < 0.001, respectively).

In the case of uropathogenic strains, we found a statistically significant association between the presence of the *irp2*, *ybtS* and *fyuA* genes and susceptibility to fosfomycin (p = 0.017, p = 0.024, and p = 0.024, respectively). Similarly, we found significant associations between the presence of the *uge* gene and susceptibility to aztreonam, cefepime, ceftazidime, chloramphenicol, ciprofloxacin and trimethoprim-sulfamethoxazole (p = 0.002, p = 0.008, p < 0.001, p = 0.004, p = 0.018 and p = 0.043, respectively). On the other hand, we found that the strains lacking the *iucA* gene were more susceptible to imipenem (p < 0.001) and chloramphenicol (p = 0.013), whereas the strains lacking the *iroN* gene were more susceptible to piperacillin/tazobactam (p = 0.001).

Among the respiratory strains, we found a statistically significant association between the presence of the *iucA* gene and susceptibility to imipenem (p = 0.036), piperacillin/tazobactam (p = 0.034), amoxicillin/clavulanate (p = 0.021), aztreonam (p = 0.006), cefepime (p = 0.036), ciprofloxacin (p = 0.004) and trimethoprim-sulfamethoxazole (p = 0.036). Similarly, we found a significant association between the presence of the *rmpA* gene and susceptibility to piperacillin/tazobactam (p = 0.022), amoxicillin/clavulanate (p = 0.025), aztreonam (p = 0.031) and ciprofloxacin (p = 0.022). The presence of the *uge* gene was correlated with susceptibility to aztreonam (p = 0.005) and ciprofloxacin (p = 0.012). However, strains lacking the *ybtS*, *irp2* or *fyuA* genes were more susceptible to gentamicin (p = 0.047 in all cases). Finally, strains carrying the *ybtS* gene were more resistant to imipenem (p = 0.039).

For the group of strains isolated from blood, we found a significant association between the presence of the *uge* gene and susceptibility to amoxicillin/clavulanate (p = 0.001), aztreonam (p < 0.001), cefepime (p < 0.001), ceftazidime (p < 0.001), chloramphenicol (p = 0.024), ciprofloxacin (p < 0.001), gentamicin (p = 0.003) and trimethoprim-sulfamethoxazole (p = 0.012). Nevertheless, strains lacking the *iucA* or *ybtS* genes were more susceptible to chloramphenicol (p = 0.024 and p = 0.016). Similarly, strains lacking the *irp2* gene were more susceptible to cefepime (p = 0.044) and gentamicin (p = 0.044). Finally, strains carrying the *irp2* gene were more resistant to aztreonam (p = 0.027).

## Discussion

*K. pneumoniae* causes a wide range of infections and uses different virulence factors to colonize and spread in the human body. The increasing antimicrobial resistance of this bacteria in recent years is of great concern to the scientific community.

In this study, we characterized a collection of 127 clinical *K. pneumoniae* strains isolated from urine, the respiratory tract, and blood and compared these strains according to antimicrobial resistance and the presence of a wide variety of virulence factors.

Overall, we found that more than 40% of isolates were classified as MDR and almost half of these were from urine. The data from the 2017 European Antimicrobial Resistance Surveillance Network report showed that at the European Union and the European economic area level, more than one third of *K. pneumoniae* isolates were resistant to at least one of the antimicrobial groups under regular surveillance. The report showed resistance percentages for fluoroquinolones (31.5%), followed by third-generation cephalosporins (31.2%), aminoglycosides (24.1%), and carbapenems (7.2%) (European Centre for Disease Prevention and Control (ECDC), 2018) similar to the general percentages observed in this study.

Urine isolates showed the highest overall antibiotic resistance (64.91%), closely followed by strains isolated from blood (63.64%) and the respiratory tract (51.35%). Uropathogenic isolates made up the majority of the ESBL-producing strains. Compared to the multicenter study carried out for Garcia-Fernandez et al., our strains showed a higher prevalence of ESBL and lower carbapenemase production. This previous study reported a prevalence of 23.1% of ESBL-producing *Klebsiella* spp. and 20% of carbapenemase-producing *Klebsiella* (García-Fernández et al., 2019). This is of great concern because some antibiotic resistance genes are carried by mobile genetic elements that facilitate horizontal genetic exchange and promote the spread of antimicrobial resistance genes within and between species. Their characterization in future studies could help us to expand our research. On the other hand, multidrug resistance has been associated with increased health care costs, longer hospital stays, and high mortality rates. Moreover, nowadays there are few new alternatives for effective treatments.

Urine strains also showed the highest resistance rates to fosfomycin (24.56%). Fosfomycin trometamol is one of the antibiotics proposed for treating uncomplicated UTIs, and its consumption has increased by 20.7% since 2012 in Spain, according to the Joint Inter-agency Antimicrobial Consumption and Resistance Analysis report (Alonso Herreras et al., 2018). Sorlozano et al. showed that ESBL-producing *Klebsiella* had low susceptibility rates to fosfomycin ranging between 40 and 78% (Sorlozano et al., 2014). Therefore, it is important to consider this antibiotic in the persistent monitoring and surveillance of antimicrobial resistance.

Regarding virulence, hypermucoviscous pathotypes, genes for adhesins, iron uptake and transport, toxins, and biofilm formation ability are some of the factors that *K. pneumoniae* employs in pathogenesis. Although many studies define hypervirulent *K. pneumoniae* (hvKp) as string test positive, it has been demonstrated that not all hvKp strains are hypermucoviscous (Fu et al., 2018). The study carried out by Russo et al. proposed the *rmpA* and *iucA* genes as biomarkers for distinguishing between hypervirulent (hvKp) and the classical (cKp) *K. pneumoniae* pathotypes (Russo et al., 2011). *rmpA* is a plasmid-located virulence factor gene that regulates the synthesis of capsular polysaccharides  (Wang et al., 2020), and *iucA* is involved in the biosynthesis of the siderophore aerobactin (De Lorenzo and Neilands, 1986). These genes were significantly associated with the hvKp-phenotype in our study, in which seven of the 17 (41.18%) hypermucoviscous strains carried both genes. On the other hand, two more strains were found, *icuA*+, *rmpA*+ and HMV-, five were classified as *icuA*+, *rmpA*- and HMV- and ten were classified as *icuA*-, *rmpA*- and HMV+. This suggests that other regulatory mechanisms may be involved in the expression of the hypermucoviscous phenotype. Thus, considering both the string test and the hypervirulence-related genes *iucA* and *rmpA*, 24 strains (18.9%) were classified as hypervirulent in the present work. In terms of antimicrobial resistance, hvKp have been classified as susceptible or as ESBL-producer strains (Harada and Doi, 2018). We found 13 strains classified as susceptible and nine as ESBL-producers, similar to the studies previously described. Of the other two strains with an *icuA*-, *rmpA*- and HMV+ phenotype, one was classified as resistant to fosfomycin and the other as resistant to more than three categories of antibiotics.

Other hypervirulence-associated genes, such as *iroBCDN, iutA, iuacABCD*, *ybt* and the *clb* locus, may be encoded in the chromosome as part of integrative conjugative elements (Arato et al., 2021). The hypervirulent phenotype confers important properties to bacteria for pathogenesis, such as capsule polysaccharide production, phagocytosis resistance, dissemination and systemic infection in the host (Xu et al., 2021). Early identification of hvKp strains would allow better understanding of the infection and the implementation of more effective antimicrobial treatment. Likewise, it is mandatory to avoid the possible dissemination of these pathotypes by implementing barriers between patients, with precautions based on virulence likely being as important as those based on antimicrobial resistance.

Among siderophores, enterobactin (encoded by *ent* genes), aerobactin (encoded by *iuc* genes), salmochelin (encoded by *iro* genes), and yersiniabactin (encoded by *ybt* genes, regulated by *irp* genes, and the receptor is encoded by the *fyuA* gene) are the most representative in *K. pneumoniae*. Siderophores are indispensable to obtain the iron necessary for the growth and replication of bacteria. They scavenge iron from host transport proteins. Enterobactin is produced by all *K. pneumoniae*, but its mechanism may be disabled by human lipocalin-2 (Lcn2), which has an affinity for enterobactin and elicits an inflammatory response. In contrast, yersiniabactin eludes binding to Lcn2, thereby avoiding the inflammatory response and promoting bacterial growth (Lam et al., 2018). As expected, we found the *entB* gene in all the strains tested in agreement with another study (Fatima et al., 2021). Besides, the higher prevalence of the siderophore-encoded *ybtS* and *fyuA* genes in the respiratory tract isolates compared to the urinary and blood strains confirmed that yersiniabactin is an important virulence factor for the pulmonary infection, as reported by previous studies (Yan et al., 2016).

The capsule-associated genes *wabG*, (encoding capsule), *uge* (encoding capsule lipoprotein) and *ycfM* (promoting external membrane protein) are highly involved in virulence by promoting infection through resistance to phagocytosis. We observed the presence of the *uge* (uridine diphosphate galacturonate-4 epimerase) gene in the majority of the isolates from urine, showing a statistically significant difference compared to the other clinical isolates. It has been demonstrated that *K. pneumoniae* strains lacking the *uge* gene are less virulent and less capable of causing UTI, pneumonia or sepsis. Mutations in the *uge* gene reduce the colonization ability of *K. pneumoniae* in experimentally induced urinary infections (Regué et al., 2004; Candan and Aksöz, 2015; Remya et al., 2019). However, the occurrence of the *uge* gene in *K. pneumoniae* has varied widely in different studies. Remya et al. found a prevalence of 48.6% harboring the *uge* gene. Its distribution in different clinical specimens was blood (50%), urine (48.8%), exudates (48.4%), and respiratory secretions (47.3%). El Fertas-Aissani et al. observed the presence of the *uge* gene in 84.6% of urinary tract strains and all the blood and respiratory isolates (El Fertas-Aissani et al., 2013). Although the *uge* gene has previously been described as essential for *K. pneumoniae* virulence, future research may provide further evidence for its specific association with UTIs.

Among the toxins tested, two of the strains were positive for the *clbA* and *clbQ* genes, two of the genes encoding colibactin. Colibactin is a recently described toxin that has been associated with colorectal cancer in humans because it causes DNA damage in host cells. Colibactin has been found in *E. coli*, but its occurrence in other species is steadily increasing. Acquisition of the *pks* locus, which synthesizes this toxin, has been linked to intestinal colonization and mucosal invasion by *K. pneumoniae* (Strakova et al., 2021).

Interestingly, one of the strains isolated from the respiratory tract was positive for all the virulence genes tested. Two other strains were positive for almost all the virulence genes except those encoding colibactin. These three strains were susceptible to all antimicrobial agents, and were classified as serotype K1, non-biofilm-forming, or weak biofilm-forming strains and showed high serum resistance, which protects the bacteria from phagocytosis (Hennequin and Robin, 2016). Although more research is needed on the influence of the acquisition of antibiotic resistance mechanisms by the bacteria on fitness cost and the relation with virulence, we found a scenario in which the greater the virulence, the lesser the antimicrobial resistance. Indeed, we found a correlation between the presence of some virulence factor genes and susceptibility to most of the antibiotics tested.

The genes encoding ESBL are carried by plasmids that may also harbor other genes, including some virulence factor genes. In previous studies, three virulence factors (adhesin CF29K, aerobactin or mucoid phenotype) were found in ESBL-producing *K. pneumoniae* strains. (Hennequin and Robin, 2016). In our study, we found the same association between *fyuA*, *irp2* or *ybtS* genes and ESBL-producing strains. The *ybt* carried by the FIB_K_ plasmid is a novel mechanism for *ybt* mobilization, Currently, many FIB_K_ plasmids also contain antimicrobial resistance transposons, which may indicate the coexistence of virulence and resistance genes in the same mobile genetic element (Lam et al., 2018).

Regarding biofilm, almost half of biofilm-forming strains, including weak, moderate and strong, were isolated from urine and the difference from the other clinical sources was statistically significant. *K. pneumoniae* has been identified as the second most prevalent pathogen in UTIs (Liu et al., 2020), and the ability to form biofilms on indwelling urethral catheters is one of the most common conditions, affecting thousands of people worldwide each year. Recurrence of catheter urinary tract infections after antibiotic treatment could be caused by re-colonization by bacteria that survived in the biofilm (Stahlhut et al., 2012). Besides, biofilms present higher resistance to phagocytosis and higher antimicrobial resistance compared to planktonic bacteria (Trautner and Darouiche, 2004). Nonetheless, truly effective strategies to prevent or counteract biofilm-related infections remain elusive.

## Conclusion

We found that isolates from urine were more resistant to antimicrobials and were more ESBL-producers, and more biofilm-formers compared to respiratory and blood strains. Our study also correlated the presence of the *uge* gene with UTIs and the presence of yersiniabactin with respiratory tract infections. In addition, although we found an inverse relation between antimicrobial resistance and virulence, the acquisition of mobile genetic elements could promote not only the spread of antimicrobial resistance genes but also that of virulence genes that evolve toward pathotypes considered to be more virulent. The increasing coexistence of these two conditions is of particular concern as it can lead to untreatable and invasive *K. pneumoniae* infections. Active surveillance not only for antimicrobial resistance but also for virulence determinants is imperative to avoid the transmission and spread of hypervirulent or extensively resistant strains.

## Data Availability Statement

The original contributions presented in the study are included in the article/**Supplementary Material**. Further inquiries can be directed to the corresponding author.

## Author Contributions

Conceptualization, SS and VB. Methodology, VB, YG, MT, RO, and CR. Statistical analysis, VB. Writing-original draft preparation, VB and SS. Writing—review and editing, VB, CR, YG, and SS. All authors contributed to the article and approved the submitted version.

## Funding

This work was funded by Planes Nacionales de I+D+iI2008‐2011/2013‐2016 and Instituto de Salud Carlos III (PI19/00478), Subdirección General de Redes y Centros de Investigación Cooperativa, Ministerio de Economía y Competitividad, Spanish Network for Research in Infectious Diseases (REIPI RD12/0015/0013 and REIPI RD16/0016/0010) co‐financed by European Development Regional Fund “A way to achieve Europe” and operative program Intelligent Growth 2014‐2020. ISGlobal is a CERCA center from the Generalitat of Catalunya and a Severo Ochoa Center (Spanish Ministry of Science, Innovations, and Universities). VB has a grant from Ministerio de Ciencia, Tecnología e Innovación (Colombia).

## Conflict of Interest

The authors declare that the research was conducted in the absence of any commercial or financial relationships that could be construed as a potential conflict of interest.

## Publisher’s Note

All claims expressed in this article are solely those of the authors and do not necessarily represent those of their affiliated organizations, or those of the publisher, the editors and the reviewers. Any product that may be evaluated in this article, or claim that may be made by its manufacturer, is not guaranteed or endorsed by the publisher.
